# PCNA appears in two populations of slow and fast diffusion with a constant ratio throughout S-phase in replicating mammalian cells

**DOI:** 10.1038/srep18779

**Published:** 2016-01-13

**Authors:** Patrick J. M. Zessin, Anje Sporbert, Mike Heilemann

**Affiliations:** 1 Institute of Physical and Theoretical Chemistry, Goethe-University Frankfurt, Frankfurt/Main, Germany; 2 Advanced Light Microscopy, Max-Delbrück Center for Molecular Medicine, Berlin, Germany

## Abstract

DNA replication is a fundamental cellular process that precedes cell division. Proliferating cell nuclear antigen (PCNA) is a central scaffold protein that orchestrates DNA replication by recruiting many factors essential for the replication machinery. We studied the mobility of PCNA in live mammalian cells using single-particle tracking in combination with photoactivated-localization microscopy (sptPALM) and found two populations. The first population which is only present in cells with active DNA replication, showed slow diffusion and was found to be located in replication foci. The second population showed fast diffusion, and represents the nucleoplasmic pool of unbound PCNA not involved in DNA replication. The ratio of these two populations remained constant throughout different stages of S-phase. A fraction of molecules in both populations showed spatially constrained mobility. We determined an exploration radius of ~100 nm for 13% of the slow-diffusing PCNA molecules, and of ~600 nm for 46% of the fast-diffusing PCNA molecules.

DNA replication is a central process in the cell cycle and is orchestrated by a large number of proteins that assemble to a complex machinery^1^. The replication of the eukaryotic genome occurs during S-phase and requires the activation of 30,000–50,000 replicons (patches of DNA replicated from one origin). Upon activation of each origin of replication, two replication forks are assembled at the unwound DNA and progress in opposite directions. A key protein in eukaryotic replication is proliferating cell nuclear antigen (PCNA), a 30 kDa protein which acts as DNA scaffold for many essential proteins involved in replication that are unable to bind to DNA directly^2^. At the core of the replication machinery, PCNA forms a sliding clamp around the DNA, which was reported to be a ring-like homotrimer loaded by replication factor C or a double-homotrimer^3^,^4^. At each replication fork the assembly of several PCNA trimers is necessary for the simultaneous synthesis of the leading strand (about 100–200 kb) and the discontinuous synthesis of the many short Okazaki fragments (150–250 bp)^5^ of the lagging strand.

PCNA is ubiquitously distributed in the nucleus during non-S phase, and during replication assembles into microscopically visible clusters of varying sizes called replication foci (RF)^6^,^7^. Characteristic patterns for RF cluster are found in early, mid and late S phase. Each RF consists of several active replicons in close spatial proximity, with each replicon containing two replication forks with several PCNA molecules. In early S-phase many small clusters of RF are observed throughout the nucleus while in late S-phase fewer but larger clusters of active RF accumulate^8^. At the molecular level, the assembly of new RFs requires either recycling of PCNA from nearby replication forks or recruitment of PCNA molecules from the nucleoplasmic pool to the replication machinery. Using modified nucleotides and fluorescence labeling, these clusters of RF were visualized and found to colocalize with sites of nascent DNA synthesis^9^. It was found that the majority of PCNA molecules do not take part in DNA replication, as only 30% of the PCNA were localized in replication foci^10^. The dynamics of PCNA inside and outside of RF cluster were studied with fluorescence recovery after photobleaching (FRAP). An average diffusion coefficient of 11–15 μm^2^/s was determined in nuclei of replicating cells^11^. Other studies revealed that PCNA, unlike other proteins involved in replication, shows only little turnover at RF but a rapidly diffusing nucleoplasmic pool in S phase and non-S phase nuclei^12^. Transition from early to adjacent later replicons within one RF cluster seems to occur by disassembly of PCNA from replication forks into a rapidly diffusing nucleoplasmic pool from where PCNA is recruited to newly activated, nearby replicons^13^. The importance of PCNA for proliferation-related functions is reflected in the constantly high expression level in transformed cell lines like HeLa, with only a 2–3 fold increase in the S-phase^14^, and the significantly lower expression level found in non-cancer cells^4^.

The spatial organization and the dynamics of proteins in cells can be investigated at the molecular level using advanced imaging techniques such as single-molecule localization microscopy (SMLM)^15^ and single-particle tracking^16^,^17^,^18^. For example, mechanistic steps in eukaryotic transcription^19^,^20^,^21^,^22^ as well as replication in fission yeast^23^ were studied at the single-molecule level. Here, we present the first single-molecule study on the dynamics of PCNA in replicating and non-replicating nuclei of mammalian cells. We fused PCNA to the photoswitchable protein mEos2 and generated a cell line stably expressing the construct. We recorded single-molecule trajectories of PCNA in live cells. Profiting from the combination of photoactivation and single-molecule tracking, we were able to record large numbers of trajectories per cell. From these trajectories, we calculated the diffusion coefficient and confinement radius. We found two distinct populations of PCNA, with a ratio remaining constant between both populations throughout different stages of S-phase. For both the slow and the fast population, a fraction of PCNA with a confined diffusion was found and the radius of confinement determined.

## Methods

### Construction of mEos2-PCNA and mEos2-NLS

cDNA of human PCNA was purchased (Open Biosystems, Huntsville, AL) and fused to mEos2 into a CMV promotor driven backbone (C2, Clontech, Palo Alto, CA). A glycine-rich linker sequence (GEGQGQGQGPGRGYAYRS), which was reported to be necessary for cell line generation^24^, was inserted as a spacer between mEos2 and PCNA. As a reference sample for free nuclear diffusion, a second plasmid was constructed with mEos2 fused to a nuclear localization sequence, NLS (GACCCCAAGAAGAAGCGCAAGGTG)^25^,^26^, which has no other reported biological function.

### Cell culture, aphdidicolin incubation and generation of a stable cell line

For transient transfection, HeLa cells (300194, Cell Line Service, Eppelheim) were seeded in 8-well chamber slides (Sarstedt, Nürnbrecht) and grown in RPMI without phenol red complemented with 1% GlutaMAX (Gibco/Invitrogen, Grand Island, NY), 5% fetal bovine serum (Biochrom AG, Berlin) and 25 mM HEPES at 37 °C, 5% CO_2_. 24 hours after seeding, cells were transiently transfected with the mEos2 plasmids using the transfection reagent FugeneHD (Promega Corporation, Madison, WI). In some experiments, DNA replication was stopped by adding 10 μg/ml aphidicolin for at least 30 minutes, according to published protocols^27^.

For generation of a stable cell line, mEos2-PCNA transfected HeLa cells were grown in 24 well dishes with 16 divisions per well (Cellstar Cloning Plate, Cat.-No. 704 160, Greiner Bio-One) for 3 weeks in media containing 400 μg/ml G418 (Geniticin, Sigma-Aldrich, St. Louis, MO). Monoclonal colonies formed and were allowed to expand until they covered a complete division. For clonal cell line selection, 20 μl of trypsin was applied to divisions containing cell colonies and incubated for 5 minutes. Each monoclonal colony was then separately transferred to 6 well plates, expanded and visually screened for moderate levels of mEos2 fluorescence and the appearance of PCNA replication pattern. Out of three positive clonal cell lines, one was chosen for this study. Cells stably expressing mEos2-PCNA were seeded into 8-well chamber slides with 170 μm thick glass bottom (Sarstedt) and imaged 24 to 48 h later.

### Image acquisition (single particle tracking photo-activated localization microscopy, sptPALM)

Single-molecule tracking was performed on a custom-built microscope as described elsewhere^28^. The microscope was equipped with a custom-built heating stage with auto-feedback loop to monitor and control sample temperature. To identify S-phase sub-stages (early, mid, late and non S-phase), diffraction-limited wide-field images of cells expressing mEos2-PCNA were obtained by averaging 100 images of the green, unconverted form of mEos2 (λ_ex_ = 488 nm) acquired at very low laser power. For single-molecule tracking of mEos2-PCNA, 20,000 frames with an exposure time of 20 ms were acquired by stochastically photoconverting subsets of mEos2 molecules in the field of view with 488 nm and reading out the fluorescence emission with 561 nm. The irradiation intensity was experimentally adjusted for optimal balance between good signal-to-noise ratio and photobleaching of mEos2. Typical irradiation intensities were 0.1 kW/cm^2^ for both, 488 nm and 561 nm (the intensity of light passing the objective was measured, as well as the illumination area). To minimize potential cell damage mEos2 was photoactivated at 488 nm rather than at 405 nm, as UV light illumination was reported to inhibit DNA synthesis^29^.

### Analysis of single-molecule tracking data

Single-molecule image stacks were analyzed with custom-written software^17^,^18^. A combination of wavelet segmentation and simulated annealing algorithms was used to extract localizations of single emitters from the image and to generate a trajectory. For each molecule which was observed for at least 160 ms (8 frames), the slope of an affine regression line fitted to the first four MSD (mean squared displacement) values that were calculated for the time intervals τ = 20, 40, 60 and 80 ms was determined. The diffusion coefficient (D) was calculated using the Einstein-Smoluchowski equation for two-dimensional diffusion:


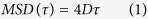


Trajectories which showed a confined diffusion were further approximated with a mono-exponential function to calculate the radius r_conf_ of the confinement area. One- and two-dimensional histograms of D and r_conf_ were plotted (Origin 9.1G, Origin Lab Corporation, MA). Using a Gaussian function, the peak values and sigma of the logarithmic distributions were determined.

Determining diffusion coefficients from single-molecule trajectories has two fundamental limitations: (i) the slowest diffusion coefficient that can be measured is determined by the localization precision; (ii) the fastest diffusion coefficient that can be measured is determined by the spatial threshold which is applied to group single-molecule localizations to a trajectory. The spatial localization precision was determined on fixed samples using a nearest neighbor analysis^30^ to σ_XY_ = 22 ± 6 nm. For moving fluorophores, this value might drop by about 80% due to velocity linked PSF blurring^31^. The lower observation limit of D is directly linked to the localization precision: molecules which explore an area smaller than the squared spatial resolution (~(2.35 · σ_XY_)^2^ = 2.7 ∙ 10^−3^ μm^2^) during 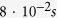 (this is the maximum time period interval, which was used to calculate the diffusion coefficient from MSD plots, see above) cannot be resolved. With equation (1) this results in a D_MIN_ of:


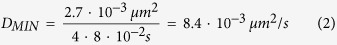


Trajectories with diffusion coefficients below D_MIN_ were therefore considered immobile. To avoid artifacts from connecting different molecules, a maximum distance threshold of 0.8 μm between subsequent single-molecule localizations was applied. This consideration allows determining D_MAX_ to:


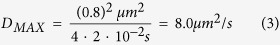


Molecules that diffuse faster than D_MAX_ are thus underrepresented.

## Results

### PCNA shows two modes of mobility

Replication of the eukaryotic genome occurs in spatially distinct clusters termed replication foci (RF)^7^. By establishing a stable HeLa cell line expressing very low levels of mEos2-PCNA, we could ensure that expression of the tagged PCNA has no influence on the cell cycle distribution and progression of cells through S-phase (Supplementary Fig. S1A/B). Live HeLa cells expressing mEos2-PCNA were pulse-labeled with the thymidine analogue BrdU to show that PCNA tagged with the fluorescent protein colocalizes with sites of ongoing DNA replication (Supplementary Fig. S1C). Even under conditions of higher PCNA expression levels due to transient transfection, no significant influence of the construct on the DNA replication was detected by incorporation of BrdU (Supplementary Fig. S2, S3).

By applying generally low light intensities and by photoconverting mEos2 with 488 nm instead of 405 nm (see Materials and Methods) imaging conditions were set such that light-induced stress was minimized. We investigated cellular stress and potential effect on DNA replication by monitoring active DNA synthesis through measuring the incorporation of EdU during and also after the sptPALM image acquisition (Supplementary Fig. S4). To do so, cells were either during or up to 48 min after end of the sptPALM image acquisition directly on the microscope stage exposed to media containing 10 μM EdU. Using the stored position of the motorized stage, cells could be relocated after the labeling of EdU with Alexa Fluor 647. The amount of EdU incorporated revealed no difference between cells which were imaged with sptPALM and those which were not, indicating that selected image acquisition conditions do not impair ongoing DNA synthesis.

**Figure 1:**
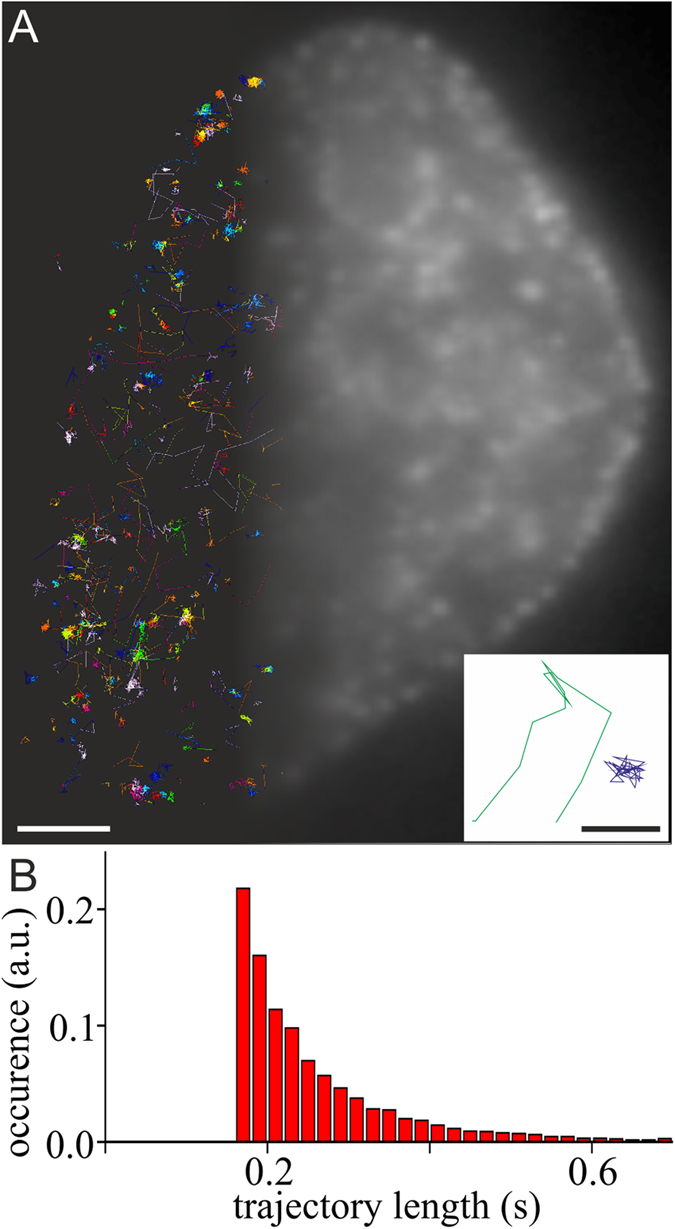
Single-molecule tracking of mEos2-PCNA in live replicating HeLa cells. (**A**) Cells stably expressing mEos2-PCNA were manually screened for replication foci pattern using the green fluorescence emission of mEos2 (top-right). Tracking of PCNA in living cells reveal different modes of PCNA mobility (top-left) (scale 2 μm). Two exemplary tracks (D_green_: 2.1 μm^2^/s; D_blue_: 0.0015 μm^2^/s) are shown in the inset (scale 500 nm). (**B**) Histogram of the trajectory length of individual PCNA molecules tracked for at least 160 ms (8 frames) (data from 18 cells, 7435 trajectories).

**Figure 2:**
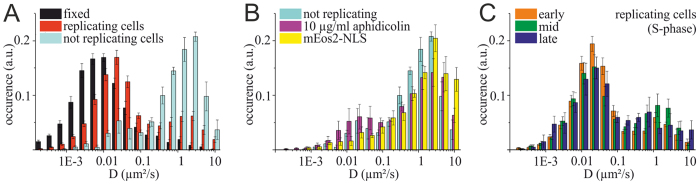
Diffusion coefficient distribution of mEos2-PCNA in replicating and not replicating HeLa (stable cell line). (**A**) Two populations of PCNA can be distinguished by their diffusion coefficient. The larger population of PCNA exhibits a low diffusion coefficient (peak 0.02 μm^2^/s) in cells showing typical patterns of replication (S-phase). In cells without replication foci (non-S-phase) the majority of PCNA molecules exhibit a high diffusion coefficient (peak 1.5 μm^2^/s) (cells analyzed: 6 fixed cells (3411 tracks); 18 cells with replication foci (7435 tracks); 3 cells without replication foci (554 tracks)). (**B**) Diffusion coefficient distribution of mEos2-PCNA in cells without replication patterns (non S-phase) and cells treated with 10 μg/ml aphidicolin to stop DNA replication. For comparison, cells were transfected with mEos2 fused to a nuclear localization sequence (NLS) confirming the unbound status of PCNA molecules with D > 0.1 μm^2^/s (3 cells without replication foci, 554 tracks; 5 cells treated with aphidicolin, 4526 tracks; 5 cells transfected with mEos2-NLS, 135 tracks). (**C**) Diffusion coefficient distributions for cells in early, mid and late S-phase exhibit a high similarity of the ratios of the slow and fast PCNA population (cells analyzed: 8 cells in early S-phase (3493 tracks); 7 in mid S-phase (3295 tracks); 3 in late S-phase (647 tracks)) (error bars represent s.e.m.).

We then used single-particle tracking to investigate the dynamics of PCNA in live mammalian nuclei in S phase (Fig. 1) and non S-phase and found two distinguishable modes of PCNA mobility, one slow and one fast population (Fig. 2). We next determined the distribution of diffusion coefficients from trajectories of single PCNA molecules in replicating cells displaying RF pattern of different S-phase stages and non-replicating cells without any visible RF. We found two well-separated populations for both stages (S and non S-phase) of the cell cycle (Fig. 2A). For 18 replicating cells, we found the majority of PCNA (69 ± 4% (s.e.m.) with D < 0.1 μm^2^/s) in a population peaking at a diffusion coefficient of 1.95 ∙ 10^−2^ μm^2^/s (s.e.m range: 1.87 ∙ 10^−2^–2.03∙ 10^−2^ μm^2^/s), which reveals a substantial mobility compared to the apparent diffusion coefficient peak of immobile PCNA in fixed samples (0.74 ∙ 10^−2^ μm^2^/s (s.e.m range: 0.72 ∙ 10^−2^–0.76 ∙ 10^−2^ μm^2^/s), Fig. 2A) as well as the theoretical lower boundary defined by the localization precision (0.84 ∙ 10^−2^ μm^2^/s, equation (2); see Material and Methods). A smaller fraction of PCNA molecules in replicating cells (31 ± 4% (s.e.m.) with D > 0.1 μm^2^/s) exhibited a faster motion peaking at a diffusion coefficient of 1.29 μm^2^/s (s.e.m range: 1.12–1.49 μm^2^/s).

In non-replicating cells, we found the majority of PCNA molecules in a population of fast diffusion (82 ± 6% (s.e.m.) with D > 0.1 μm^2^/s) peaking at 1.48 μm^2^/s (s.e.m range: 1.30–1.68 μm^2^/s). A smaller fraction of PCNA molecules in a slower population with a diffusion coefficient (15 ± 8% (s.e.m.) with D < 0.1 μm^2^/s) peaking at 3.02 ∙ 10^−2^ μm^2^/s (s.e.m range: 2.70 ∙ 10^−2^–3.38 ∙ 10^−2^ μm^2^/s) exists. This may indicate that a small fraction of PCNA in non S-phase nuclei exists that is involved in complex formation or binding to structures independent of DNA replication.

We reason that the slower population represents PCNA molecules actively participating in DNA replication inside RF, whereas PCNA molecules with higher diffusion coefficient are localized outside of RF and do not interact specifically with chromatin. As shown in supplementary Fig. S5A/B structures resembling replication foci known from diffraction-limited microscopy are formed by PCNA molecules of the slow population. Trajectories belonging to the fast population show a much bigger area of exploration.

In order to corroborate this finding, we repeated single-molecule tracking of PCNA in cells treated with aphidicolin. Aphidicolin reversibly stops DNA replication by inhibiting DNA polymerase alpha and delta^27^, which results in the almost complete disassembly of PCNA from the RF into the nucleoplasm, with only a few PCNA remaining associated to RF^32^. We found that in cells treated with 10 μg/ml aphidicolin, PCNA was predominantly found in the population of fast diffusion, overlapping well with the histogram of diffusion coefficients found for non-replicating cells (Fig. 2B). PCNA molecules with small diffusion coefficient exhibiting a small exploration area disappear when DNA replication is blocked by incubation of cells with aphidicolin (Supplementary Fig. S5 A/B). Also here, we find a fraction of PCNA molecules with a slower diffusion coefficient similar as in non-replicating cells, which might indicate for a subfraction of PCNA involved in processes independent of active DNA replication.

### Slow and fast population of PCNA stay in balance throughout early, mid and late S-phase

The number and distribution of replication foci clusters is changing throughout different stages of S-phase^8^,^33^. Furthermore, typical patterns for early, mid and late S-phase were reported^24^. In order to determine whether the sub-stages of S-phase and the number of active RF affected the mobility of PCNA we investigated the diffusion behavior of PCNA in cells grouped into the three sub-phases of S-phase: early, mid and late (Supplementary Fig. S1D). Notably, we found very similar distributions for the diffusion coefficient of PCNA (Pearson’s r (15) > 0.91; p > 0.001; see Supplementary Fig. S6) in all three sub-phases of replicating cells (Fig. 2C). The majority of PCNA molecules (early: 75 ± 8%; mid: 63 ± 9%; late: 66 ± 9% (s.e.m.)) showed a slow diffusion (D < 0.1 μm^2^/s), in all S-phase stages and is likely engaged in DNA replication. A second and smaller population of PCNA molecules showed fast diffusion, and we speculate that these PCNA molecules constitute the soluble pool. Although pattern and number of RF is different in S-phase sub-stages, the ratio between bound PCNA engaged in replication and mobile PCNA in the nucleoplasmic pool is almost constant.

### A subset of PCNA molecules in both populations shows confined movement

**Figure 3:**
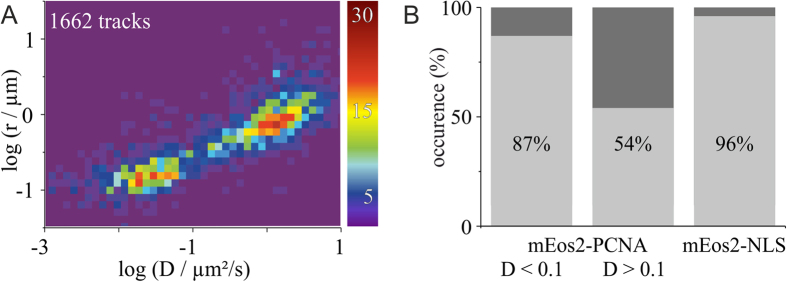
PCNA molecules are not only distinguishable by diffusion coefficient, but also by radius of confinement. (**A**) Both slow and fast PCNA populations exhibit confined (dark grey bars) and free (light grey bars) diffusion. 13% (658 out of 5254 trajectories) of PCNA molecules belonging to the slow-diffusing population (D < 0.1 μm^2^/s) and 46% (1004 out of 2181 trajectories) belonging to the fast-diffusing population (D > 0.1 μm^2^/s) exhibit confined diffusion. The radius of confinement was calculated for each single trajectory, and 2D-histogrammed with the diffusion coefficient. PCNA molecules of the slow population show a peak at r = 0.1 μm, whereas the fast population peaks at r = 0.6 μm (total number of tracks: 1662). (**B**) The majority of PCNA molecules diffuses without constrains. 87% of the slowly diffusing (D < 0.1 μm^2^/s) and 54% of the fast diffusing (D > 0.1 μm^2^/s) mEos2-PCNA molecules reveal an unconfined motion. Trajectories of mEos2 monomers fused to a nuclear localization sequence (NLS, 30 kDa) reveal predominantly free diffusion (96%).

Single-particle tracking does not only allow calculating the diffusion coefficient, but also distinguishing free diffusion, active transport, confinement to micro-domains or complete immobilization^34^. In case of confined diffusion, the radius of the confinement area (exploration area) can be directly extracted from the MSD plot^18^. We determined the confinement radius for PCNA molecules in- and outside of replication foci. A double-logarithmic plot of the confinement radius against the diffusion coefficient reveals two PCNA populations (Fig. 3A). We found a confinement radius of 110 ± 16 nm (s.d.) for the slow PCNA population, which is larger than the average radius of individual RF pulse-labeled with BrdU throughout S-phase measured with super-resolution techniques^33^,^35^. For the fast-diffusing PCNA population, a confinement radius of 600 ± 100 nm (s.d.) was found, which is several times smaller than the radius of an average HeLa nucleus^36^.

The majority of PCNA molecules diffuses without constrains. However, a small percentage (13%) of the slow-diffusing PCNA molecules, but surprisingly nearly half (46%) of the fast-diffusing PCNA molecules, showed confined diffusion (Fig. 3B). We determined the diffusion mode of mEos2-NLS and found that the majority (96%) of freely diffused in the cell nucleus (Fig. 3B).

## Discussion

The mobility of a protein in a cell is influenced by interactions with other biomolecules or the existence of spatially confined compartments. Advanced light microscopy techniques can probe these interactions and visualize such compartments, and were previously used to study the kinetics of DNA replication and transcription^23^,^37^. PCNA is a key protein in eukaryotic DNA replication, and its mobility has been intensively studied^11^,^23^,^38^. In this study, we investigated the mobility of individual PCNA molecules in mammalian cells and determined both diffusion coefficients as well as diffusion mode in different stages of the cell cycle. We found two well-separated populations of PCNA in S-phase (Fig. 1, Supplementary Fig. S5), exhibiting peak diffusion coefficients of 0.02 μm^2^/s and 1.29 μm^2^/s, respectively (Fig. 2).

The existence of two populations of PCNA was already reported in FRAP studies. The diffusion of PCNA in the whole nucleus (11–15 μm^2^/s^11^) was found to be remarkably faster compared to the diffusion coefficient inside replication foci (4 × 10^−4^ μm^2^/s; calculated as described in^39^ on data shown in^12^). Although FRAP is a method that averages over a large number of molecules, the results are in good agreement with the average values of the diffusion coefficient distribution found in this study using single-molecule tracking. The apparent discrepancy of the diffusion coefficients of fast-diffusing PCNA is explained by specific experimental constraints of single-molecule tracking that lead to an underrepresentation of fast molecules (see materials and methods for a detailed discussion). Although this limitation could in principle be avoided by using shorter integration times, this would in turn reduce the spatial resolution of the experiment.

From our results we concluded that slow-diffusing PCNA molecules are directly engaged in DNA synthesis whereas fast-diffusing PCNA represents the soluble nucleoplasmic pool. The majority of trajectories of slow-diffusing PCNA molecules occur in small areas whose size and pattern resembles replication foci (Supplementary Fig. S5). This observation is supported by the absence of the majority of the slow population in cells without visible replication pattern (non S-phase), as well as in cells that were treated with aphidicolin to stop active replication (Fig. 2, Supplementary Fig. S5). In addition to previous studies on PCNA mobility, a small population of slow-diffusing PCNA molecules was found in non S-phase cells and cells with impaired DNA synthesis (Fig. 2). This population is not found for mEos2-NLS, which has no biological function in the nucleus. PCNA is reported to be involved in many processes besides DNA replication, e.g. DNA repair, chromatin remodeling, and cell cycle control by interaction with a multitude of proteins^2^. However, most of these interactions occur in S-phase or at G1/S-phase transition and are coupled to the function of PCNA as DNA scaffolding protein. The potential functions of PCNA without DNA interaction are less well investigated, although interaction partners involved in immune response, translation, proteolysis are published (for an overview see^40^).

The natively homogeneous distribution, as well as the disappearance of the slow diffusing population of PCNA in non-replicating and replication-impaired cells, clearly shows that the expression of mEos2-PCNA did not lead to artificial clustering caused by the fluorescent protein.

DNA replication is a well regulated process and deregulation is linked to cancer and cell death^41^. The regulation occurs on many levels, which makes it more robust on the one hand, and on the other hand allows the cell to react in a flexible way to various sources of disturbance. PCNA is often found highly upregulated in cancer cells, and it was speculated that the number of accessible PCNA molecules has a major impact on DNA replication and thus might be subject of regulation as well^42^. This strongly suggests a need to tightly control the amount of available PCNA over the course of replication, in order to avoid replicative stress and subsequent DNA damage. Our data strengthens this hypothesis: the ratio between active (slow) and inactive (fast) PCNA remains almost constant during different states of S-phase (Fig. 2). Interestingly, the number of RF was reported to change during the course of S-phase decreasing from about 6800 in early S to about 2000 in late S-phase^33^. To cope with a need for different amounts of PCNA in different stages of S-phase (higher amounts in early S, lower amounts in late S –phase), the total amount of PCNA molecules (bound and unbound) might be a subject of regulation in the course of S-phase.

The peak diffusion coefficient determined for the slow population of PCNA (0.02 μm^2^/s) is higher than the apparent diffusion coefficient of PCNA in fixed cells (0.008 μm^2^/s). It is also higher than the average diffusion coefficient that was reported for chromatin itself (10^−4^–10^−3^ μm^2^/s^43^); (note that this value is not accessible to the experimental approach used in this work). PCNA therefore shows a measurable motion when it takes part in DNA replication. As the influx of PCNA molecules from the nucleoplasm into the replication foci is rather low^13^, recycling of PCNA within clusters of RF visible with diffraction-limited microscopy (which have a variable size in the different S-phase stages) may underlie this motion. Some degree of mobility is a necessity for PCNA molecules participating in DNA synthesis for both the constant assembly of new replications forks at nearby newly activated RF, and the synthesis of multiple Okazaki fragment within established RF clusters.

Studies using different super-resolution methods^33^,^35^ reported an average size of 125–150 nm for RF (ranging from 40 nm to 210 nm) that appears to be conserved throughout S-phase. This implies that larger RF clusters, especially found in later stages of S-phase, are composed of smaller, individual RF. Interestingly, a subset of the slow diffusing PCNA molecules shows a spatial confinement within a radius of 110 ± 16 nm (Fig. 3), which is nearly twice as large as the average radius of the replication foci measured with super-resolution microscopy^33^,^35^. Considering the localization precision of 22 nm, the area for recruitment and recycling of PCNA molecules might therefore be larger than the size of replication foci measured by diffraction-unlimited imaging methods. One possible explanation could be the participation of PCNA in post-replicative processes such as chromatin modification, remodeling and chromosome assembly via interaction with proteins such as DNMT1, CAF1 or HDAC^44^.

The distribution of diffusion coefficients of the fast population of mEos2-PCNA (~60 kDa) is comparable to the one of free mEos2-NLS monomers (~30 kDa, Fig. 2). This indicates at a rather unhindered movement of PCNA inside the nucleus - and in case of the constrained molecules, the identified subdomains -, without relevant interaction with the chromatin. This also argues against the existence of larger, pre-assembled complexes of PCNA with other proteins involved in DNA replication in the fast fraction of PCNA molecules. The high mobility of PCNA may ensure that the high number of origins and replication forks assembled throughout S-phase is always supplied with sufficient PCNA molecules.

A detailed analysis of single-molecule trajectories of fast-diffusing PCNA revealed that about half of the trajectories show a confined movement (Fig. 3). This constrained diffusion was not observed for mEos2-NLS. The confinement radius of 0.6 μm is several times smaller than the radius of the nucleus. It was suggested that the nucleus has a non-random spatial organization, which imposes restrictions on diffusing molecules equal to material with fractal or porous geometry^45^,^46^. One fascinating example for compartmentalization inside the nucleus is the organization of chromosomes into distinct domains called chromosome territories^47^,^48^. The average radius of chromosome territories in human cells was reported to be in the range of 400 to 800 nm^49^,^50^, which fits strikingly well with the calculated confinement radius of the PCNA population not involved in active DNA replication. The confinement of a fraction of fast-diffusing PCNA might thus be connected to the question how chromosome territories keep their exclusive organization^51^. Gaps or channels between the chromosome territories, as suggested by the chromosome territory-interchromatin compartment model^52^,^53^, might explain the unhindered diffusion of the remaining fraction of fast-diffusing PCNA population. At this point, this interpretation is rather speculative and requires experiments about the coincidence of chromosome territories with areas of PCNA confinement.

In summary, we investigated the dynamics of PCNA in the nuclei of live mammalian cells using single-molecule tracking. We found two modes of PCNA mobility, one slow and one fast population. We found evidence for a global regulation towards a stable ratio between fast diffusing and a slow PCNA population involved in DNA replication throughout different stages in S-phase. Furthermore, we found that a fraction of PCNA engaged in DNA replication is restricted to an area which exceeds the typical size of individual replication foci determined by diffraction-unlimited imaging techniques. We speculate that the confined movement of nearly half of the fast diffusing PCNA molecules is due to nuclear sub-compartments, such as chromosome territories.

## Additional Information

**How to cite this article**: Zessin, P. J. M. *et al.* PCNA appears in two populations of slow and fast diffusion with a constant ratio throughout S-phase in replicating mammalian cells. *Sci. Rep.* **6**, 18779; doi: 10.1038/srep18779 (2016).

## Supplementary Material

Supplementary Information
