# Supplementary material for: PCNA appears in two populations of slow and fast diffusion with a constant ratio throughout S-phase in replicating mammalian cells
Source: Sci Rep. 2016 Jan 13;6:18779. doi: 10.1038/srep18779 (PMC4725372; doi:10.1038/srep18779)
Supplement: Supplementary Information [file srep18779-s1.pdf]

PCNA appears in two populations of slow and fast diffusion with a constant ratio throughout S-phase in replicating mammalian cells

Patrick J. M. Zessin[1], Anje Sporbert[2, *], Mike Heilemann[1, *]

[1]Institute of Physical and Theoretical Chemistry, Goethe-University Frankfurt, Frankfurt/Main Main, Germany

[2]Advanced Light Microscopy, Max-Delbrück Center for Molecular Medicine, Berlin, Germany

*correspondence: heilemann@chemie.uni-frankfurt.de, asporb@mdc-berlin.de

Supplementary material

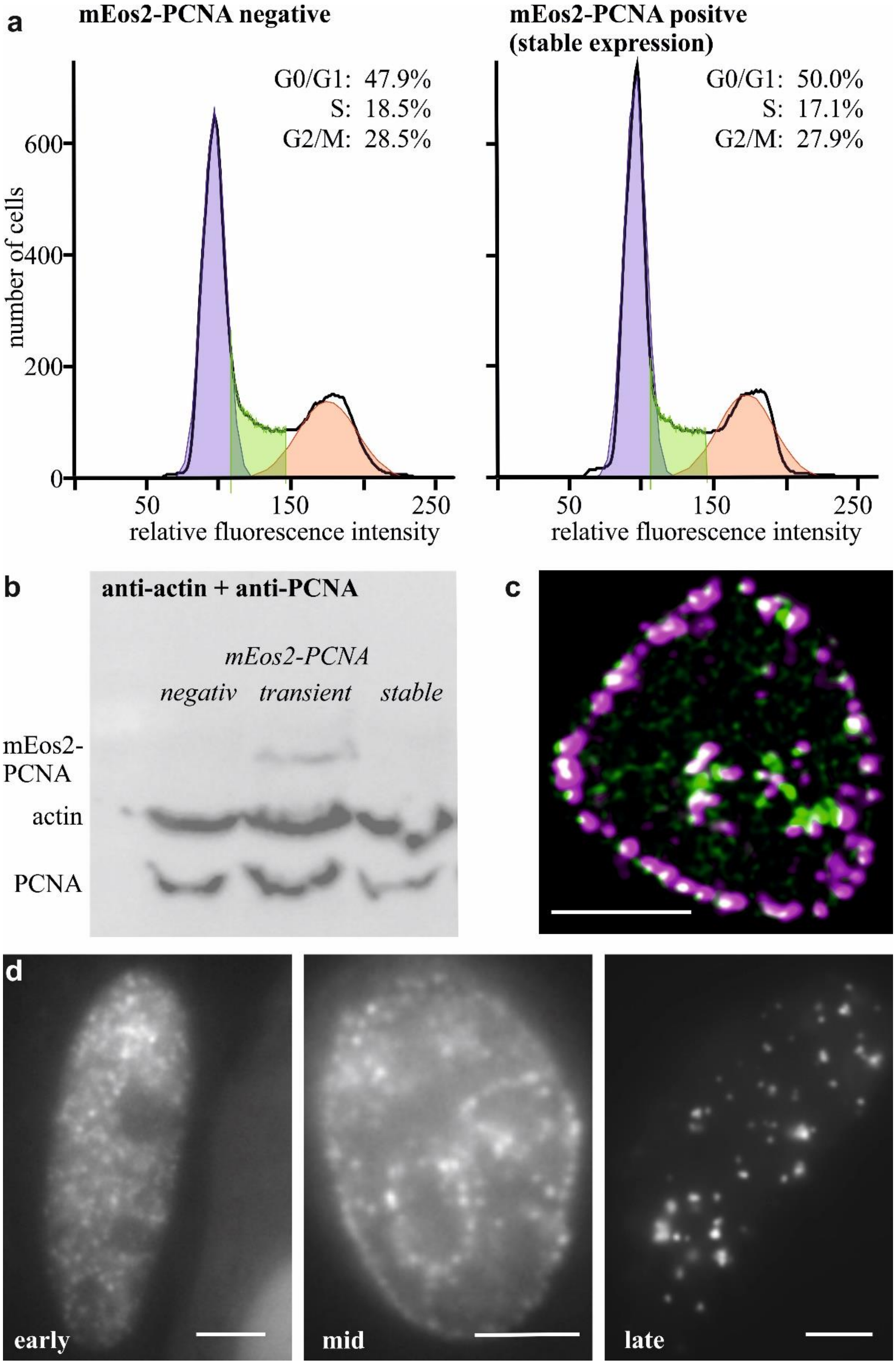

FIGURE S1 Characterization of mEos2-PCNA and stable expressing HeLa cell line.

(a) Cell cycle distribution of parental HeLa cells (mEos2-PCNA negative) and the mEos2-PCNA HeLa cell line (mEos2-PCNA positive) analyzed by flow cytometry. For both, very similar amounts of cells are found in G0/G1, S-phase and G2/M for both indicating that low expression levels of mEos2-PCNA in the cell line have no influence on the cell cycle. **(b) Expression of mEos2-PCNA** in transiently transfected HeLa cells (about 90% transfection efficiency) and in the stable expressing HeLa cell line analyzed by SDS-PAGE/Western Blot. Untreated HeLa cells were used as negative control. Actin (42 kDa) was detected to assess the overall protein concentration in all three samples. In all three samples, endogenous PCNA (30 kDa) was detected. In transiently transfected cells a second band of mEos2-PCNA (60 kDa) becomes visible indicating the right size of the PCNA fusion protein. In the stably expressing cell line, mEos2-PCNA expression remains below the detection limit of the western blot**.** For more experimental details see supplementary methods below **(c) Colocalisation of mEos2-PCNA with BrdU labeling sites of ongoing DNA replication.** HeLa cells transiently expressing mEos2-PCNA were pulse labeled with 10 µM BrdU for 30 minutes. Confocal microscopy shows colocalization (white) of PCNA (green) and BrdU (magenta) in replication pattern of a typical mid S-phase nucleus. Scale: 5 µm **(d) mEos2-PCNA expressed in replicating HeLa cells forms S-phase specific cluster of replication foci (RF) pattern**. Preceding sptPALM experiments, widefield images of mEos2-PCNA positive cells were acquired using the green fluorescent state of mEos2 (excitation: 488 nm; detection: 520 nm). Cells were grouped as early, mid and late S-phase cells based on their RF pattern as reported in the literature [1]. Scale: 5 µm

(a)

Sample	*Cell count*	*BrdU +*	*mEos2 +*	*BrdU +/mEos2 +*
mEos2-PCNA	893	28.2%	19.8%	6.8%
mEos2-H2B	338	26.0%	44.1%	10.4%
Mock	511	25.1%	n.a.	n.a.
Negative	336	35.4%	n.a.	n.a.

(b)

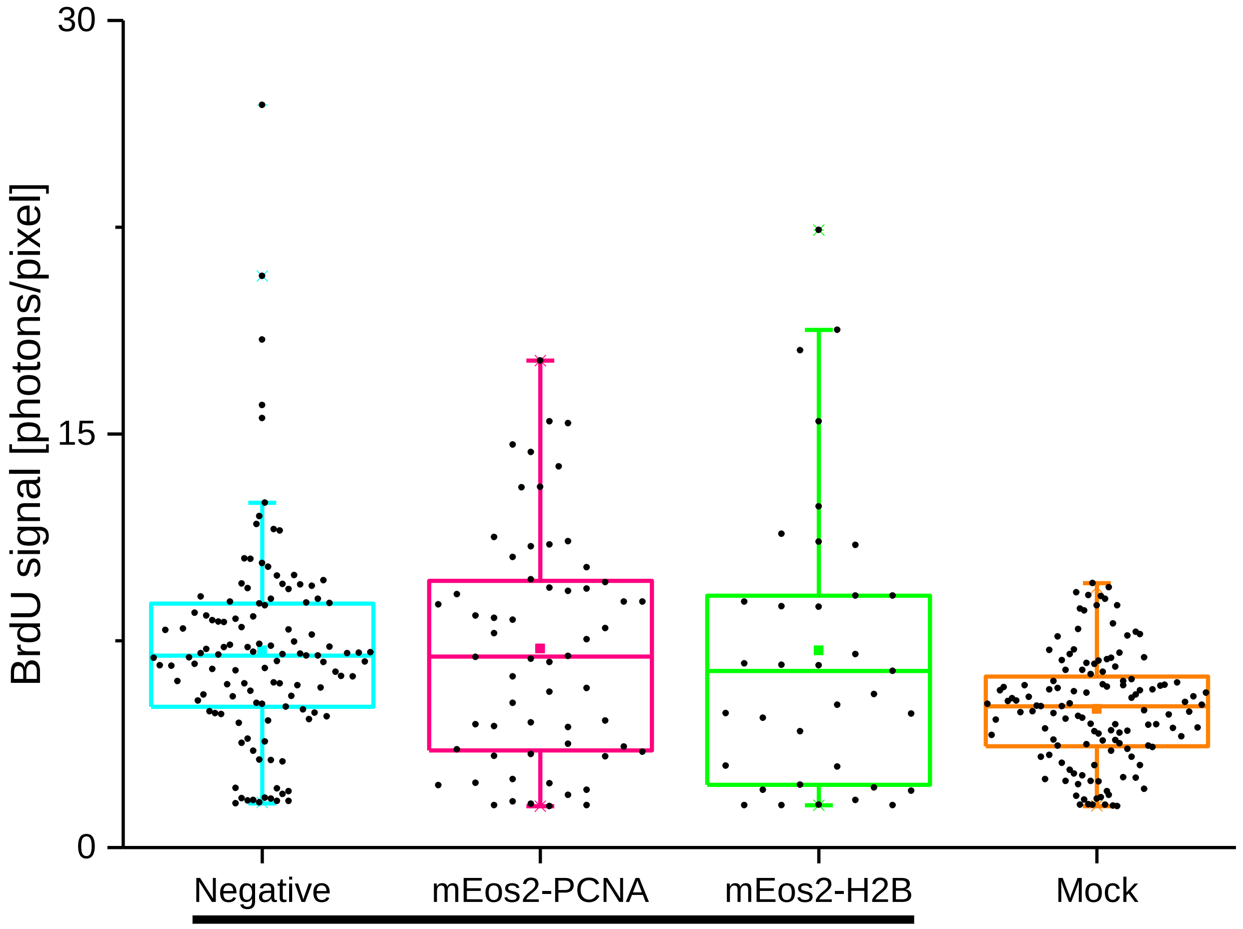

FIGURE S2 Percentage of BrdU incorporating cells und amount of BrdU incorporation are similarin transfected and untransfected cells

(a) HeLa cells expressing mEos2-PCNA or mEos2-H2B (as control) were pulse-labeled with BrdU for 30 minutes. BrdU was immunostained with Alexa Fluor 647. Nuclei were identified using DAPI as reference staining and analyzed for fluorescence emission of Alexa Fluor 647 and mEos2. Cells showing a higher average intensity than 1.5 photons per pixel were counted as positive (percentage given with respect to the total number of DAPI stained nuclei). Note that the percentage of replicating cells (BrdU +) does not differ for transfected and mock-transfected cells as well as for cells containing different mEOS2 fusion proteins (PCNA and H2B, double positive cells). This clearly shows that the transfection of cells with mEos2-PCNA does not alter the cell cycle. (b) The average fluorescence intensity of Alexa Fluor 647 inside of nuclei was measured and compared. The difference in

the average intensities of Alexa Fluor 647 and thus the amount of BrdU incorporation into the DNA between untransfected cells (negative), cells positive for mEos2-PCNA and mEos2-H2B was found to be non-significant (Tukey-Kramer test, depicted with black bar). In conclusion, the incorporation of mEos2-PCNA into the replicon of replicating cells does not affect DNA synthesis.

(a)

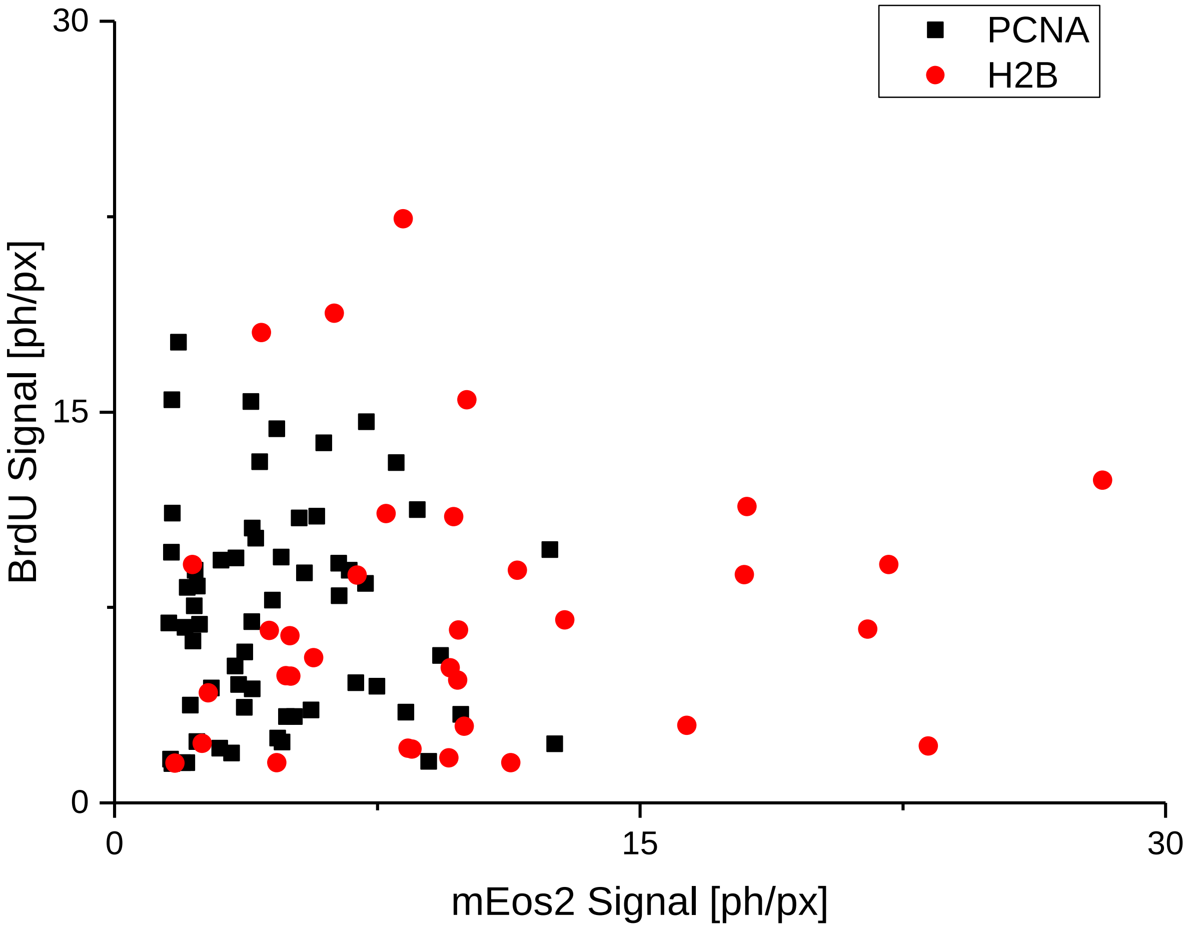

(b)

Correlation pair	*correlation value*	*p value*
mEos2-PCNA and BrdU	0.005	0.97
mEos2-H2B and BrdU	0.036	0.84

FIGURE S3 The transfection level of mEos2-PCNA or mEos2-H2B does not affect replication.

(a) Scatterplot of average fluorescence intensity of BrdU labeled with Alexa Fluor 647 and mEos2 in double positive cells (intensity is given in photons per pixel). (b) Regression analysis of both BrdU and mEos2-PCNA/-H2B signal revealed no correlation (correlation value < 0.04). The level of mEos2-PCNA expression of (mEos2 signal strength) has therefore no influence on the incorporation of BrdU into DNA.

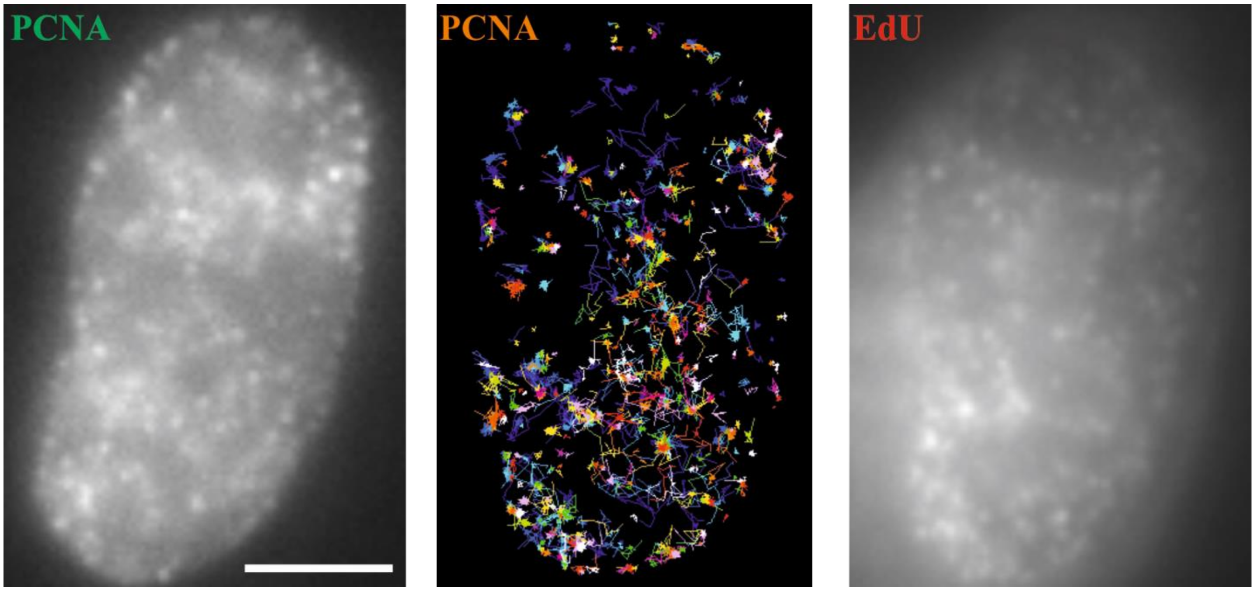

FIGURE S4 sptPALM does not inhibit DNA replication.

Green fluorescence of mEos2-PCNA transiently expressed in HeLa cells (left panel, scale bar 5 µm) was imaged immediately before sptPALM acquisition of 20,000 frames (400 s). EdU (10 µM) was added to the cells at the start of sptPALM acquisition to monitor ongoing DNA synthesis during the time of acquisition. Every acquired mEos2-PCNA track longer than 8 frames is displayed and color-coded according to its time of occurrence (mid panel). Directly after acquisition, the cell was fixed and EdU incorporated into nascent DNA during the time of acquisition was click-labeled by Alexa Fluor 647 azide (right panel). Using the stored position of the motorized microscope stage, the correct cell was relocated. Due to fixation and the staining procedure for EdU the morphology of the nucleus is slightly changed.

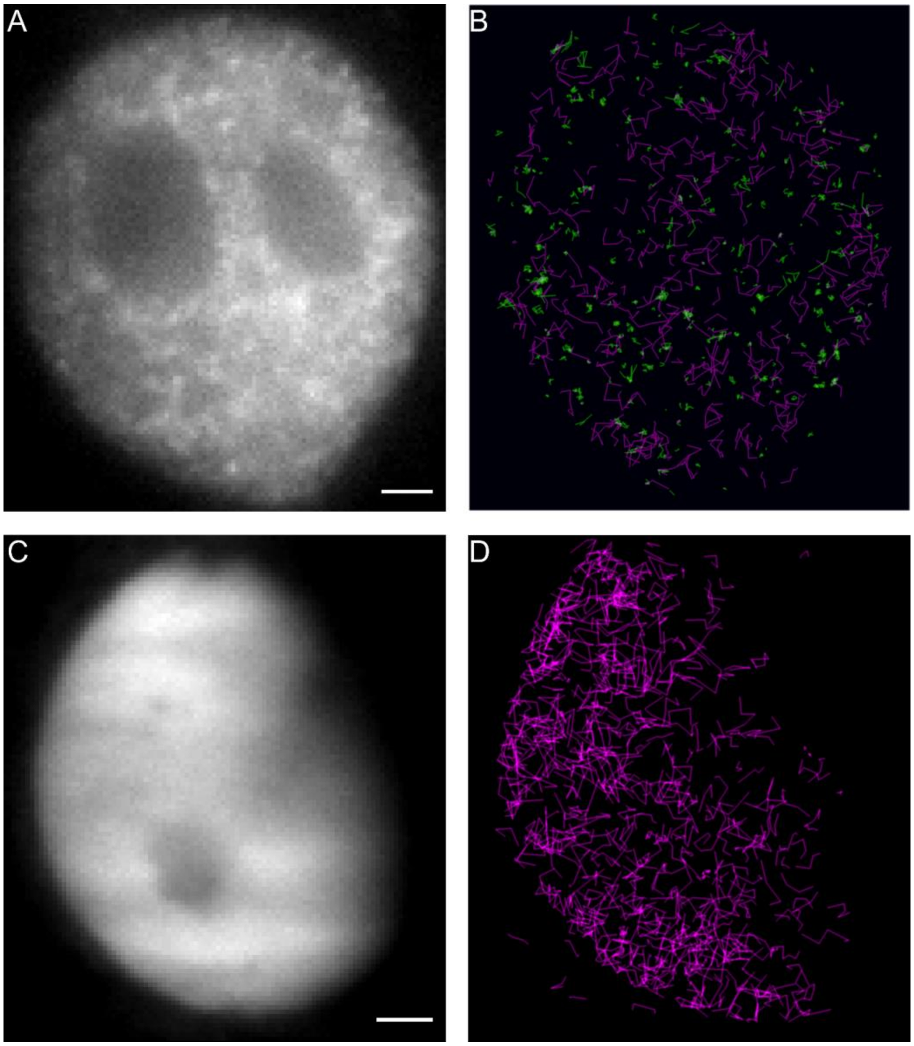

FIGURE S5 PCNA trajectories of two representative cells.

(A) HeLa cell expressing mEos2-PCNA with typical early S-phase replication pattern. (B) Trajectories of PCNA molecules tracked in (A) were colored-coded according to diffusion coefficient: green trajectories belong to the slow population ($D < 0.1$ µm²/s), magenta trajectories belong to the fast population ($D > 0.1$ µm²/s). Structures resembling replication foci known from diffraction-limited microscopy are formed by PCNA molecules of the slow population. Trajectories belonging to the fast population show a much bigger area of exploration. (C) HeLa cell expressing mEos2-PCNA treated with 10 µg/ml aphidicolin for at least 30 minutes. (D) Trajectories of PCNA molecules tracked in (C). With replication blocked by aphidicolin, trajectories of PCNA with RF sized exploration radius become absent. Scale: 2 µm.

Correlated S phase distributions	***Correlation Value***	***P Value***
Early : Mid	**0.93**	**$4.7 \cdot 10^{-08}$**
Early : Late	**0.95**	**$4.9 \cdot 10^{-09}$**
Mid : Late	**0.91**	**$2.1 \cdot 10^{-07}$**

FIGURE S6 Similarity of diffusion coefficient distributions during S phase.

The distribution of diffusion coefficients does not change significantly during the course of replication (Fig. 2C). Each D distribution of early, mid and late S-phase cells was correlated and revealed high correlation values (> 0.91).

Supplementary Methods

Western blot

HeLa cells were plated in 10 cm petri dishes (Greiner; 10^6 cells per dish). 24 h after seeding, one petri dish was transfected with mEos2-PCNA using FugeneHD (Promega). 48h after transfection, the cells in all three dishes were lysed in Laemmli sample buffer. The lysate was incubated for 10 min at 95° for protein denaturation. 5 µl of protein extract were loaded on a 12% sodium dodecyl sulfate polyacrylamide gel and separated by size with gel electrophoresis (SDS-PAGE). After electrophoresis, the proteins were blotted onto a polyvinylidenedifluoride (PVDF) membrane using the Trans-Blot Turbo System (Bio Rad) at 25 V for 30 min. To reduce unspecific antibody binding the membranes were incubated in 5% nonfat dry milk (Roth)/TBS-T (20 nM Tris; 150 mM NaCl; 0.1% Tween; pH 7.6) for 1 h. For detection of PCNA and actin, membranes were incubated with monoclonal anti-PCNA antibody (Dako, Clone PC10, M0879), 1:1000 and monoclonal anti-beta actin (8H10D10) antibody (Cell Signaling, #3700), 1:5000. As secondary antibody anti-mouse IgG peroxidase conjugate (Sigma, A2554), 1:10000, was used. All antibodies were diluted in TBS-T. Primary and secondary antibody incubation was conducted for 1h at RT. Luminescence signals were detected with the Lumi-Imager F1 (Roche) using the Clarity Western Blot ECL Substrate (Bio Rad).

Flow cytometry

Flow cytometry was used to analyze the cell cycle distribution of parental HeLa cells and mEos2-PCNA stable expressing cell line. Subconfluent cells were trypsinized, fixed with 70 % ethanol and stained with 50µg/ml propidium iodid (PI) containing 100µg/ml RNAse A. Fluorescence intensity of PI stained DNA was quantified with a LSR II flow cytometer (BD Biosciences). At least 10.000 cells were collected and histograms analyzed with the FlowJo V.10 software.

Labeling of nascent DNA

Nascent DNA was labeled by supplementing growth medium with 10 µM EdU (5-ethynyl-2-deoxyuridine, Baseclick, Tutzing) and stained with Alexa Fluor 647 as reported previously [2]. After fixation with 2% formaldehyde in PBS, cell membranes were permeabilized with 0.5 % Triton X-100 (Sigma-Aldrich) and incubated with reaction buffer (5 µM Alexa Fluor 647 azide, 100 mM HEPES pH 8.2, 1 mM $CuSO_4$, 50 mM aminoguanidine and 25 mM ascorbic acid) for 15 minutes.

To analyze the incorporation of EdU into the DNA during and after sptPALM imaging, 6 cells with early S-phase replication patterns were selected and their positions in respect to the corner of the cell culture well were stored. Using the motorized stage, all 6 cells were imaged with sptPALM sequentially for 400 s (20,000 frames). Before imaging the sixths cell (presented in Fig

S2), 200 µl medium containing 20 µM EdU (end concentration: 10 µM) was directly added to the media on stage. Directly after image acquisition of the sixths cell, 400 µl of 4% formaldehyde was added directly to the medium on stage (end concentration: 2%). The cells were incubated in formaldehyde for 10 minutes and then labeled with Alexa Fluor 647 azide as described above. Using the position list stored before the click reaction and allocating it to the reference point (corner of the well) allowed to relocate all 6 cells. Acquiring widefield images of EdU-Alexa Fluor 647 revealed no difference in intensity between cells which were imaged with sptPALM and those, which were not.

BrdU labeling and Confocal microscopy

For colocalization analysis of BrdU and PCNA, samples were transfected with mEos2-PCNA as described earlier. As a reference sample, mEos2-H2B was constructed by exchanging the PCNA sequence by the sequence of the nuclear protein H2B. 24h after transfection, cells were pulse-labeled for 30 minutes with 10 µM BrdU and directly fixed with 4% formaldehyde in PBS. BrdU was detected using a mouse anti-BrdU antibody (Becton-Dickinson) and incubation with a puffer containing DNAse I [3] to preserve mEos2 fluorescence and labeled by Alexa Fluor 647.

Cells revealing both mEos2 and Alexa Fluor 647 signal were imaged with a confocal laser scanning microscope (Leica SP8 equipped with Hybrid detectors in photon counting mode). Images were processed with the open-source software Fiji. Analysis of BrdU and mEos2 signal intensity was conducted using the Leica software LAS X.

Statistical analysis

Data statistics were acquired with Excel as described in the literature [4].

References

1 Dimitrova, D. S. & Berezney, R. The spatio-temporal organization of DNA replication sites is identical in primary, immortalized and transformed mammalian cells. *J. Cell Sci.* **115**, 4037-4051 (2002).

2 Zessin, P. J., Finan, K. & Heilemann, M. Super-resolution fluorescence imaging of chromosomal DNA. *J Struct Biol* **177**, 344-348, doi:10.1016/j.jsb.2011.12.015 (2012).

3 Sporbert, A., Gahl, A., Ankerhold, R., Leonhardt, H. & Cardoso, M. C. DNA polymerase clamp shows little turnover at established replication sites but sequential de novo assembly at adjacent origin clusters. *Mol. Cell* **10**, 1355–1365 (2002).

4 McDonald, R. H. *Handbook of Biological Statistics*. 3rd edn, (Sparky House Publishing, 2014).
